# Injectable stress relaxation gelatin-based hydrogels with positive surface charge for adsorption of aggrecan and facile cartilage tissue regeneration

**DOI:** 10.1186/s12951-021-00950-0

**Published:** 2021-07-18

**Authors:** Kai-Yang Wang, Xiang-Yun Jin, Yu-Hui Ma, Wei-Jie Cai, Wei-Yuan Xiao, Zhi-Wei Li, Xin Qi, Jian Ding

**Affiliations:** 1 Department of Orthopedic Surgery, Shanghai Jiao Tong University Affiliated Sixth People’s Hospital, NO. 600, Yishan Rd, Shanghai, 200233 People’s Republic of China; 2 Department of Orthopedic Trauma, Department of Orthopedics, School of Medicine, Renji Hospital, Shanghai Jiao Tong University, Shanghai, 200127 People’s Republic of China; 3 Department of Rehabilitation Medicine, Shanghai Jiao Tong University Affiliated Sixth People’s Hospital, NO. 600, Yishan Rd, Shanghai, 200233 People’s Republic of China; 4 Department of Orthopaedics, Shanghai Pudong Hospital, Fudan University Pudong Medical Center, No.2800 Gongwei Road, Huinan Town, Pudong, Shanghai, China

**Keywords:** Hydrogel, Polylysine, Aggrecan adsorption, Dynamic covalent bond, Cartilage tissue engineering

## Abstract

**Background:**

Cartilage injury and pathological degeneration are reported in millions of patients globally. Cartilages such as articular hyaline cartilage are characterized by poor self-regeneration ability due to lack of vascular tissue. Current treatment methods adopt foreign cartilage analogue implants or microfracture surgery to accelerate tissue repair and regeneration. These methods are invasive and are associated with the formation of fibrocartilage, which warrants further exploration of new cartilage repair materials. The present study aims to develop an injectable modified gelatin hydrogel.

**Method:**

The hydrogel effectively adsorbed proteoglycans secreted by chondrocytes adjacent to the cartilage tissue in situ, and rapidly formed suitable chondrocyte survival microenvironment modified by ε-poly-L-lysine (EPL). Besides, dynamic covalent bonds were introduced between glucose and phenylboronic acids (PBA). These bonds formed reversible covalent interactions between the cis−diol groups on polyols and the ionic boronate state of PBA. PBA-modified hydrogel induced significant stress relaxation, which improved chondrocyte viability and cartilage differentiation of stem cells. Further, we explored the ability of these hydrogels to promote chondrocyte viability and cartilage differentiation of stem cells through chemical and mechanical modifications.

**Results:**

In vivo and in vitro results demonstrated that the hydrogels exhibited efficient biocompatibility. EPL and PBA modified GelMA hydrogel (Gel-EPL/B) showed stronger activity on chondrocytes compared to the GelMA control group. The Gel-EPL/B group induced the secretion of more extracellular matrix and improved the chondrogenic differentiation potential of stem cells. Finally, thus hydrogel promoted the tissue repair of cartilage defects.

**Conclusion:**

Modified hydrogel is effective in cartilage tissue repair.

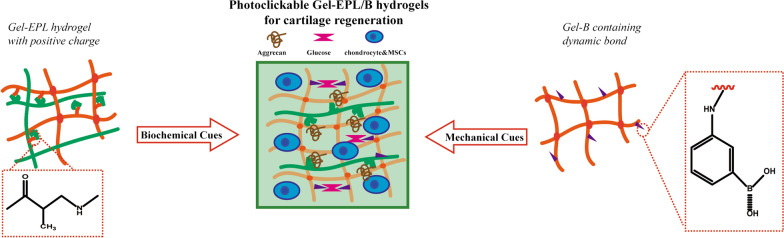

**Supplementary Information:**

The online version contains supplementary material available at 10.1186/s12951-021-00950-0.

## Introduction

Articular cartilage defect and subchondral bone degeneration of the knee joint are prevailing clinical conditions associated with knee joint dysfunction, severe pain, and disability in some cases [1]. The cartilage tissue has a complex structure and lacks blood vessels, which explains its poor spontaneous regeneration ability [2]. In articular cartilage, the extracellular matrix (ECM) mainly comprises aggrecan and type II collagen. Aggrecan comprises several chains of sulfated glycosaminoglycans, including chondroitin sulfate, which are responsible for high fixed charged density in the cartilage. Aggrecan maintains the cartilage phenotype, promotes chondrocyte proliferation and chondrogenic differentiation of bone marrow-derived mesenchymal stem cells [3]. Therefore, most biomimetic scaffolds are designed to achieve this goal. The role of the cellular microenvironment in cartilage or bone has, in the recent past, become an intense area of research. Numerous studies have proved through silane modification techniques, that cell behavioral response to various surface chemical groups [4]. For instance, modifications of the surface chemistry of material were found to affect the quantity and type of adsorbed protein [5], mesenchymal stem cell (MSC) adhesion, morphology, and the differentiation potential [6]. In another study, functionalization of hydroxyapatite with RGD improved the adhesion of osteoblasts onto the hydroxyapatite surface and promoted osteoinduction [7]. Additional reports previously suggested that it is proteins or extracellular matrix in the microenvironment rather than stem cells and other cells that have direct and rapid contact with the surface of materials. As such, the selective adsorption of components in the microenvironment more effectively influences cell fate within covalently crosslinked hydrogels through restriction of cell spreading, cell viability, and cell differentiation [8]. Loebel et al. reported that nascent protein deposition occurs immediately after administration of 3D hydrogels [8]. The study demonstrated that the nascent protein can be deposited within a day, thereby masking interfacial chemical interaction of the hydrogel. This consequently affects the differentiation behavior of cells for several weeks. Chondrocytes and bone marrow-derived mesenchymal stem cells play a key role in cartilage repair. Therefore, this study explored a cellular niche with in situ high adsorptions of extracellular matrix protein and glycosaminoglycan to improve the viability of native chondrocytes and the differentiation of MSCs.

Mechanical properties of materials and density of biomaterials regulate the distribution of new matrix proteins in extracellular space during injury [9]. New proteins are kept closer to the cells when the microenvironment becomes harder and more disconnected. A soft microenvironment, therefore, promotes the spread of the newly formed matrix to the whole structure [10]. Compelling evidence shows that the mechanical properties of biomaterials affect the fate of cells [11–13]. Chaudhuri et al. found an approach for the modulation of stress relaxation properties using alginate hydrogels. They also reported that substrate stress relaxation exerts key functions in cell biology [14]. Elsewhere, Lin et al. found that reducing the degree of crosslinking of PEGS hydrogel increases stress relaxation of hydrogels, enhances chondrocyte differentiation, maintains cartilage phenotype, and enhances extracellular matrix secretion[15]. Therefore, dynamic bonds can be introduced into hydrogels to promote cell communication within the extracellular matrix. In some studies, boronic acids as receptors or sensors for carbohydrates were found [16–18]. Phenylboronic acids (PBA) have emerged as synthetic receptors that can reversibly bind to cis-diols of glucose molecules[19, 20]. In this study, nascent ECM protein microenvironment and suitable mechanical environment were incorporated into the three-dimensional hydrogel to improve chondrocyte viability and chondrogenic differentiation ability of stem cells.

GelMA hydrogels are widely used for tissue repair as they have high biocompatibility, biodegradability, bioactivity, and diversity [21]. GelMA hydrogel is formed by introducing double bonds into gelatin polymer chains, which rapidly form hydrogels under photoinitiation. The blue light initiator Lithium acylphosphinate salt (LAP) makes the gelation process faster and the preparation process easier. It is non-toxic. GelMA hydrogel is effective in injectable hydrogel preparation and can be utilized for 3D printing molding [22, 23]. Therefore, we selected GelMA hydrogel as the base hydrogel and blue light initiator to induce gelation under blue light. Positive charges were introduced into GelMA to rapidly adsorb aggrecan extracellular matrix secreted by chondrocytes, thereby creating the best cell microenvironment. ε-poly-L-lysine (EPL), biodegradable short-chain laminin is widely used in medicine as a gene carrier, hydrogel, tissue section, and glass adhesive [24–27]. EPL promotes the adhesion, growth, and proliferation of cartilage cells, epidermal cells, and adipose-derived stem cells (ADSCs). Amino-rich EPL endowed polymer positive charges, which can electrostatically absorb aggrecan with a negative charge. In addition, a phenylboronic acid (PBA) modified GelMA hydrogel was developed to obtain reversible covalent interaction for efficient ECM deposition. This hydrogel responds to stimuli of the changing environmental condition [28, 29].

**Scheme 1:**
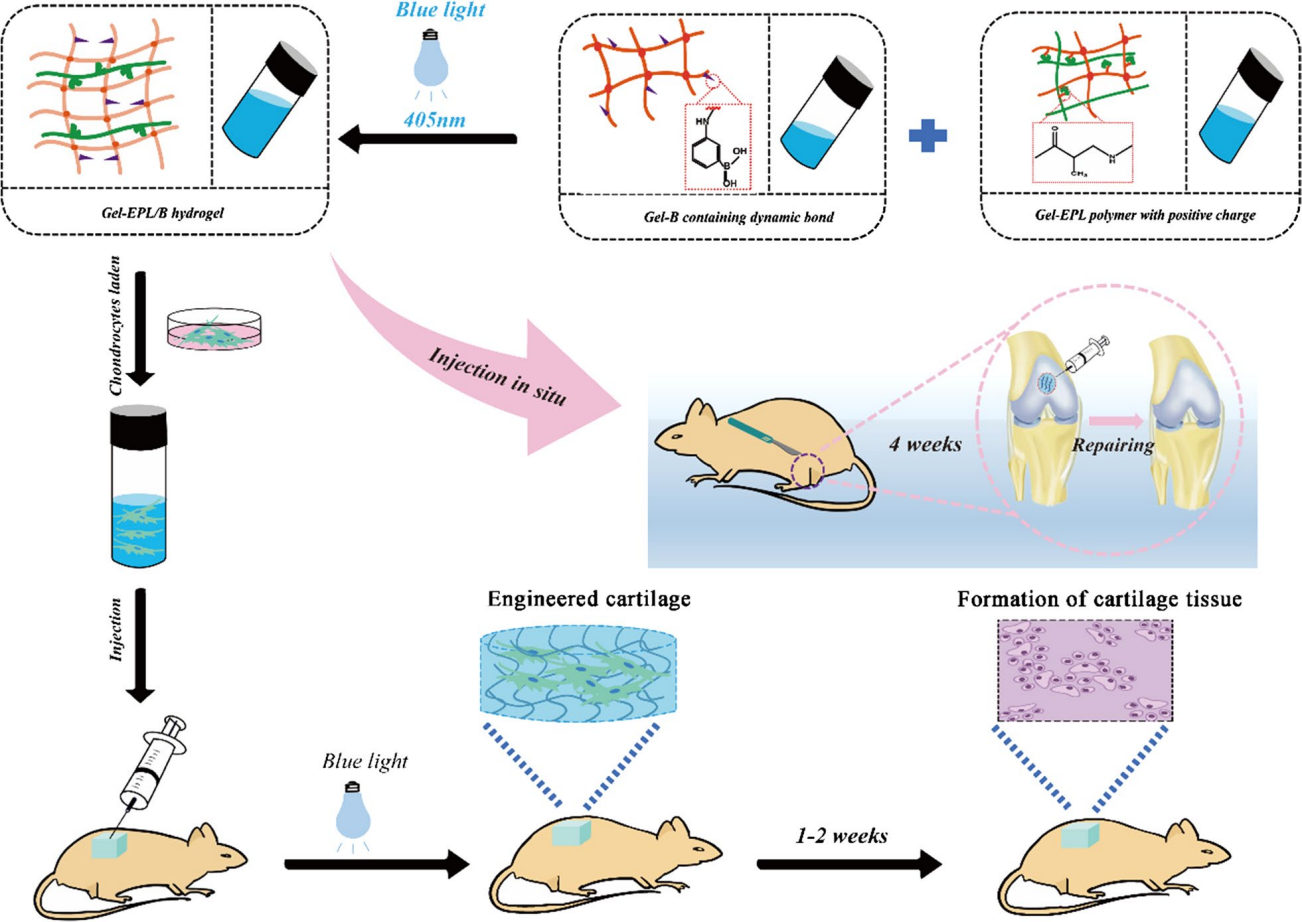
Preparation of injectable modified GelMA hydrogels as a 3D scaffold to promote cartilage tissue regeneration

With these in mind, we herein, designed and synthesized new biodegradable hydrogels which can be easily biofunctionalized for tissue regeneration. The theoretical motivation behind this approach is as follows: The surface charge of the fabricated hydrogels as chemical cues could further enhance the deposition of extracellular matrix in the cartilage. These hydrogels could also provide a stress relaxation microenvironment for cell expansion, extracellular matrix deposition, and cell to cell communication. We evaluated the interaction between glucose and phenylboronic acids (PBA) through mechanical approaches as a reversible covalent interaction between cis-diol groups on polyols and ionic boronate state of PBA [30]. The hydrogel reported here is an injectable, minimally invasive photocrosslinkable GelMA hydrogel for cartilage repair (Scheme 1).

## Materials and methods

### Materials

Gelatin methacrylamide (GelMA) (82% substitution degrees), ε-poly-L-lysine (EPL, Mn = 3500), 3-Aminophenylboronic acid (PBA), and Paraformaldehyde were purchased from Aladdin Industrial Corporation (Shanghai, China). Lithium acylphosphinate salt (LAP), Dimethylmethylene blue (DMMB), and Collagenase (246 units/mg) were purchased from Sigma-Aldrich (USA). Fetal bovine serum (FBS), phosphate-buffered saline (PBS), MEM Alpha Modification (α-MEM), and Dulbecco’s modified Eagle’s medium (DMEM) were purchased from GIBCO-Life Technologies. The TRIzol reagent was obtained from the Takara Company. The antibodies (Sox9, Col2a1, and Aggrecan) were purchased from Abcam (U.K.).

### Synthesis and characterization of gel-EPL and gel-EPL/B pre-polymer

**Fig. 1:**
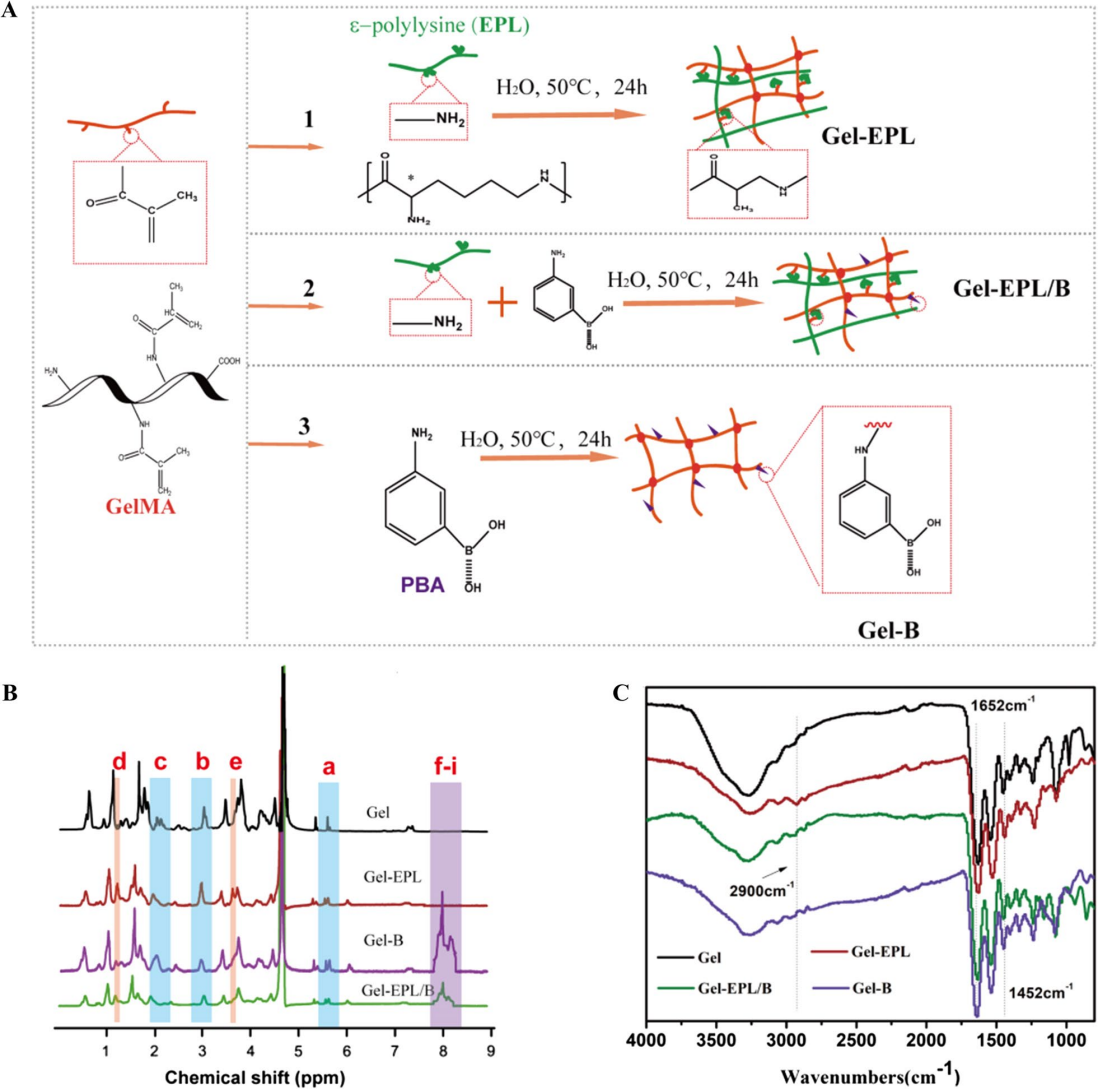
**A** Scheme showing hydrogel preparation process and the reaction mechanism. (1) Synthesis and schematic process for the formation of Gel-EPL hydrogel; (2) Synthesis and schematic process for the formation of Gel-EPL/B hydrogel; (3) Synthesis and the schematic process for the formation of Gel-B hydrogel. **B** ^1^H NMR spectra of the GelMA and modified GelMA polymers in D_2_O (Chemical structure was shown in Additional file 1: Fig. S1A). **C** The FTIR spectra of polymers

EPL-modified GelMA copolymers were synthesized by the Michael addition approach between GelMA and ε-poly-L-lysine (EPL) (Fig. 1A). Exactly 250 mg of GelMA (0.42 mmol/g methacrylamide group) was dissolved in 10 ml water. Further, 0.01, 0.006, and 0.001 mmol of EPL (Mn = 3500) were dissolved in 2.5 ml of water in nitrogen at 50 ℃. The EPL solution was gradually added to the GelMA solution and allowed to react at 50 ℃ for 24 h. Dialysis was then performed in a 3500-dialysis bag for 2 days, and the mixture was lyophilized. The products were coded Gel-EPL 0.1, Gel-EPL 0.05, and Gel-EPL 0.01. Synthesis of PBA-modified polymer was performed following a method described by Yesilyurt et al. [31]. To synthesize PBA-modified GelMA, PBA solution was mixed with GelMA solution via the Michael addition approach, similar to the synthesis of Gel-EPL. To synthesize Gel-EPL/B, 18.375 mg EPL and 0.719 mg amino-phenylboronic acid were dissolved in 2.5 ml water. The mixture was then gradually added to GelMA (250 mg) solution. After 24 h, the solution was dialyzed for three days, and then lyophilized. For the synthesis of Gel-B, only 1.438 mg PBA was added to the GelMA solution. Modification of GelMA was monitored by Fourier transform infrared spectroscopy (FTIR, Nicolet5700, USA) and Proton Nuclear Magnetic Resonance (^1^HNMR, Bruker Avance II 600, Bruker Corporation, Switzerland). The sample was dissolved in D_2_O to attain a concentration of 5 mg/ml. and the data were processed and analyzed in MestreNova NMR software. Zen3600 was used to measure zeta potential [32]. Polymers were dispersed in Ultra-pure water (10 mg/mL) and filtered through VertiPure Nylon syringe filters (0.45 μm). The filtered sample was filled into polystyrene cuvettes. The Zen3600 automatically calculated the zeta potential of electrophoretic mobility. FTIR spectra were produced using an FTIR Excalibur Series instrument (FTIR, Nicolet 6700) at a frequency range of 4000–400 cm^−1^.

### Preparation and characterization of gel-EPL and gel-EPL/B hydrogels

**Fig. 2:**
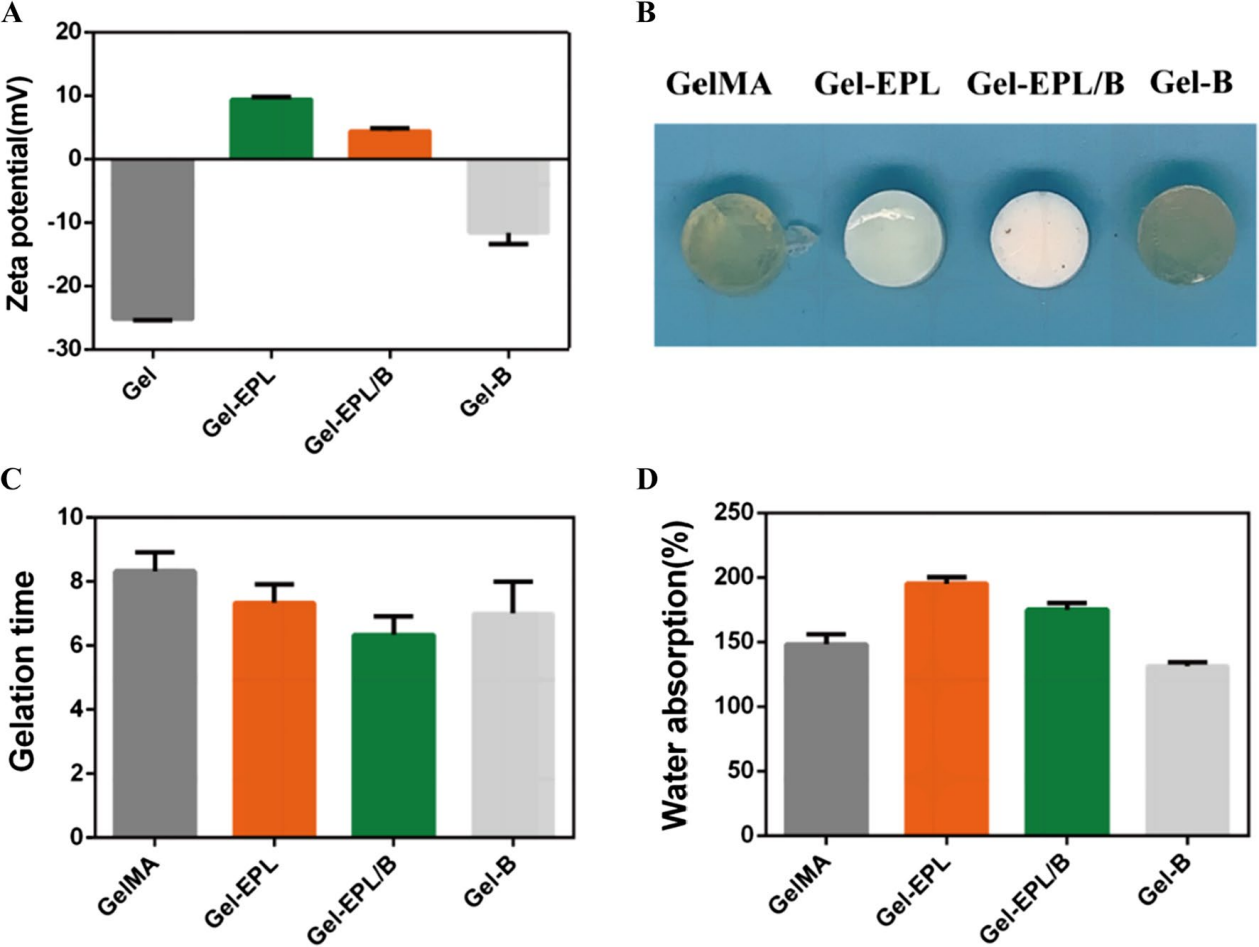
**A** Zeta potential recorded for the GelMA polymer and modified GelMA polymer. **B** Digital images of the hydrogel. **C** Gelation time(s) of different hydrogels. **D** The swelling ratio of different hydrogels

Hydrogels containing 0.1 wt.% Lithium phenyl-2,4,6-trimethyl benzoyl phosphinate (LAP) were prepared via free radical polymerization induced under blue light (405 nm, Long Wave Ultraviolet Lamp (Upland, CA)) [33]. LAP is a photoinitiator with good biocompatibility than Irgacure 2959. Mishra et al*.* had previously used blue light as an energy source to cross-link PEGDA based prepolymer formulations into hydrogels for the entrapment of NIH 3T3 fibroblasts. The entrapped cells maintained excellent cell viability and cellular activities [34]. Firstly, the polymer was dissolved in water, and the concentration of hydrogel pre-solution was 20%. The solution was then poured into a Teflon mold and placed under 405 nm light for gelation. Figure 2B shows the appearance of hydrogels. Gelation time of hydrogels was measured through the vial inverting method under blue light (405 nm). Different lyophilized scaffolds were weighed (M_0_) and incubated in PBS (pH = 7.4) for 24 h. The surface of the swollen scaffolds was then gently blotted with filter paper to remove any excess swelling agent. The scaffolds were lyophilized again and weighed to determine the dry weight (Ms). Equilibrium-swelling ratio of hydrogels was determined using a gravimetric method. Field emission scanning electron microscope (FESEM, S4800, Hitachi, Japan) was used to explore morphology of hydrogels. Compression modulus of the GelMA and modified GelMA hydrogels were measured using commercial mechanical tester (SANS CMT2503) with a 20 N load cell to determine their mechanical properties. Prepared hydrogels were soaked in high sugar DMEM (4.5 g/L) for 4 h to determine stress relaxation properties. A strain of 0.15 was applied to the samples. Stress was recorded over time during 600 s while maintaining constant strain.

### Cell culture

This study was approved by the Animal Research Committee of Shanghai Jiao Tong University Affiliated Sixth People’s Hospital. Rat bone marrow mesenchymal stem cells (rBMSCs) were isolated from bone marrow aspirates of femur obtained from four-week-old rats. Primary rBMSCs were expanded to passage 3 in growth media. Growth media containing α-MEM with 10% (v/v) FBS and 1% (v/v) penicillin–streptomycin at 37 °C in humid conditions of 5% CO_2_ were used in all experiments. Chondrocytes were harvested from Sprague–Dawley (SD) rat fresh knee cartilage tissue. Fresh knee cartilage tissue was cut into slices and digested with 0.25% trypsin supplement containing 0.02% EDTA at 37 ℃ for 30 min. Tissue slices were then digested using 0.2% collagenase II in serum-free DMEM culture medium at 37 ℃ for 6 h. Chondrocytes were harvested, counted, and seeded onto culture dishes in DMEM with 10% fetal bovine serum, 100 unit/mL penicillin, and 100 mg/mL streptomycin at 37℃ under a humid condition of 95% air and 5% CO_2_. Passage 2 chondrocytes were used in experiments.

### Cell adhesion of rBMSCS on the surfaces of hydrogels

Hydrogels (100 μL, 20%, w/v) were developed in 96-well plates through photo-crosslinking. A total of 6000 rBMSCs were then seeded on the surface of the hydrogel. After culturing for 24 h, cellular morphology was examined using a confocal laser scanning microscope (CLSM, Nikon A1R, Japan) and scanning electron microscope (SEM, S-4800, Hitachi, Japan). For fluorescent staining, cells were stained using FITC-phalloidin to visualize cytoskeleton and 40, 6-diamidino-2-phenylindole (DAPI) to visualize cell nuclei.

### Surface immobilization capability of hydrogels

Biomaterial surface chemistry affects cell–biomaterial interactions by absorbing different proteins or macromolecules such as aggrecan [35]. Each group of hydrogels was soaked in Chondroitin sulfate (CS) solution containing 1 mg/mL. The supernatant was obtained after 12 h and the CS content was measured without adsorption via the DMMB method. The percentage of adsorption was calculated. For adsorption of hydrogels on proteoglycans secreted by cells, chondrocytes were inoculated into Transwell chambers. Hydrogels were then placed in the plates. Hydrogels were taken out after 12 h and stained with alcian blue.

### Biofunctionalization of hydrogels

The regenerative potential of hydrogels was evaluated. 3D cell cultures were prepared in vitro for 2 weeks (Fig. 5A). Expression levels of chondrogenesis genes and Western blot analysis were used to evaluate the chondrogenic differentiation potential of rBMSCs cultured in different hydrogels. Chondrocytes were cultured in different groups of hydrogels to explore their metabolic activity. Two weeks later, tissues were fixed, sectioned, and stained. The secretion ability of chondrocytes in a three-dimensional hydrogel matrix was determined via fluorescent staining of proteoglycan in the matrix.

### Biocompatibility of hydrogels

GelMA, Gel-EPL, Gel-EPL/B, and Gel-B samples were subcutaneously implanted on the back of 6-week-old male C57 mice. Mice were anaesthetized using pentobarbital (40 mg kg^−1^) before implantation. After 7 days of implantation, mice were sacrificed to retrieve the samples. Hydrogels and adjacent tissues were isolated and fixed in 4% paraformaldehyde. Paraffin-embedded samples were then subjected to hematoxylin and eosin (H&E) staining. CD68 immunostaining was conducted to observe infiltrated inflammatory macrophages. All treatments and experiments of animals were performed following the guidelines for Laboratory Animal Center of Shanghai Jiao Tong University Affiliated Sixth People’s Hospital.

### Chondrocyte in vivo viability evaluation

Chondrocytes were mixed with hydrogel (100 μL, 20%, w/v) solution containing LAP (0.1%, w/v) at a concentration of 1 × 10^7^ cells/mL. Polymer/cells hydrogel was then prepared by exposing to blue light irradiation (405 nm) followed by implanting subcutaneously in nude mice (6 weeks old; 3 mice in each group). Mice were sacrificed after 2 weeks by cervical dislocation. Modified tissues were isolated, fixed in 4% paraformaldehyde, and embedded in paraffin. Embedded tissues were stained using H&E, Acan, and Collagen type II and observed using an inverted fluorescence microscope (DMi8, Leica, Germany).

### Regeneration of cartilage defects in vivo

All animals were treated according to standard guidelines outlined by the Animal Research Committee of Shanghai Jiao Tong University Affiliated Sixth People’s Hospital. Twelve male SD rats (3 rats, 6 femurs in each group) weighing 200 g on average were randomly categorized into 4 groups: (1) GelMA group, GelMA solution containing LAP (0.1%, w/v) was injected into cartilage defects (2 mm in diameter and 1 mm in depth) followed by one-minute exposure to blue light; (2) Gel-EPL group; (3) Gel-EPL/B group; (4) Gel-B. At specific time intervals, the rats were sacrificed. The distal femurs were gross examined and then fixed in 10% formalin, decalcified, embedded in paraffin, and ultimately subjected to histochemical staining (H&E, Safranin O, Collagen type II). For histological and immunohistological analysis, paraffin sections were cut into 4.5 μm. The slides were hydrated and stained with hematoxylin and eosin (H&E) and Safranin-O (Saf-O) for histological examination of cell morphology and sGAG production. The slides were prepared to detect the presence of collagens II via immunohistochemistry as previously described [15]. Visualization was based on enzymatic conversion of a chromogenic substrate, 3,3- diaminobenzidine (DAB) into a colored brown chromogen at the sites of antigen localization. The stained slides were mounted for observation using bright-field microscopy, all images were captured.

### Statistical analysis

One-way analysis of variance (ANOVA) was used to determine differences between groups at a confidence interval of 95%.

## Results

### Synthesis of gel-EPL and gel-EPL/B pre-polymer

Chemical structures of EPL-modified gelatin and PBA-modified gelatin were analyzed by ^1^H NMR. The two strong peaks at δ 5.3 and 5.6 ppm were assigned to acrylic protons and the peaks at δ 2.0 and 2.9 ppm represented the methyl function. Reduction of resonance signals of acrylic protons and significant enhancement peaks from EPL at δ 3.81, 3.10, 1.75, 1.45, and 1.27 ppm (Fig. 1B) indicated successful synthesis of Gel-EPL polymer. Two peaks at 7.8 and 8.2 ppm (b) representing protons of benzene ring were observed in the^ 1^H NMR spectrum of PBA-modified gelatin. The reaction degree of various modified GelMAs was evaluated using general ^1^H NMR procedures. In the ^1^HNMR spectrum, ethylene substratum at 5.6 ppm was compared to 2.0 ppm (–COCH3) to calculate the percentage of reactive acryloyl groups on GelMA. After the reaction of the double bond with the amino group, peaks appearing at 5.6 ppm in the GelMA spectrum decreased. With an increase of EPL, double bonds in the GelMA decreased (Additional file 1: Fig. S1B). The degree of modification for Gel-EPL,Gel-EPL/B and Gel-B was 19.2, 21.4, and 27.5%, respectively. We further assessed the chemical structures of Gel, Gel-EPL, Gel-EPL/B, and Gel-B using FITR (Fig. 1C). The large bands at 3200–3500 cm^−1^ (assigned to O–H stretching) were identified for all characterized materials. The absorption of –CH = CH2– in-plane bending vibration of GelMA presented at 1652 cm^−1^ and 1452 cm^−1^, respectively. Nearly 2900 cm^−1^ (assigned to C–H stretching) were from the EPL part. The band at 1650 cm^−1^ corresponded to the C = C in the polycyclic aromatic graphene PBA ring. However, the signal of C = O in the GelMA overlapped with the signal of C = C. In addition, Zeta potential results showed successful modification of polymers. We measured the Zeta potential to verify the realization of scaffold tunable surface charge in the polymer. The surface of unmodified GelMA polymer in double distilled water had a negative charge, and the potential was—25.1 mV (Fig. 2A). After modification with EPL, the zeta potential of Gel-EPL increased to + 9.4 mV, indicating that amino groups were successfully incorporated onto the surface. The zeta potential for PBA-modified polymer was − 12.2 mV, whereas the zeta potential for Gel-EPL/B double-modified polymer was + 4.3 mV.

Here, we performed TG (Additional file 1: Fig. S1C) and DSC (Additional file 1: Fig. S1D) analyses to evaluate the thermal properties of polymers. The thermal degradation behavior of polymers was explored via TG analysis (Additional file 1: Fig. S1C). The early mass loss occurred at about 100 °C, which could be attributed to the loss of chemically combined water. The mass loss stage was almost 300 °C and could be ascribed to polymer degradation. The final residual weight percentages were 26.2, 40.7, 42.3, and 42.8 wt% for the Gel, Gel-EPL, Gel-EPL/B, and Gel-B polymers, respectively. Gel-EPL showed lower glass-transition temperature (Tg) compared to Gel (Additional file 1: Fig. S1D). By incorporating PBA, Gel-B and Gel-EPL/B showed higher Tg due to the hard-blocks of PBA in polymers. These results demonstrated the high thermal stability of Gel and modified polymers.

### Preparation and characterization of gel-EPL and gel-EPL/B hydrogels

**Fig. 3:**
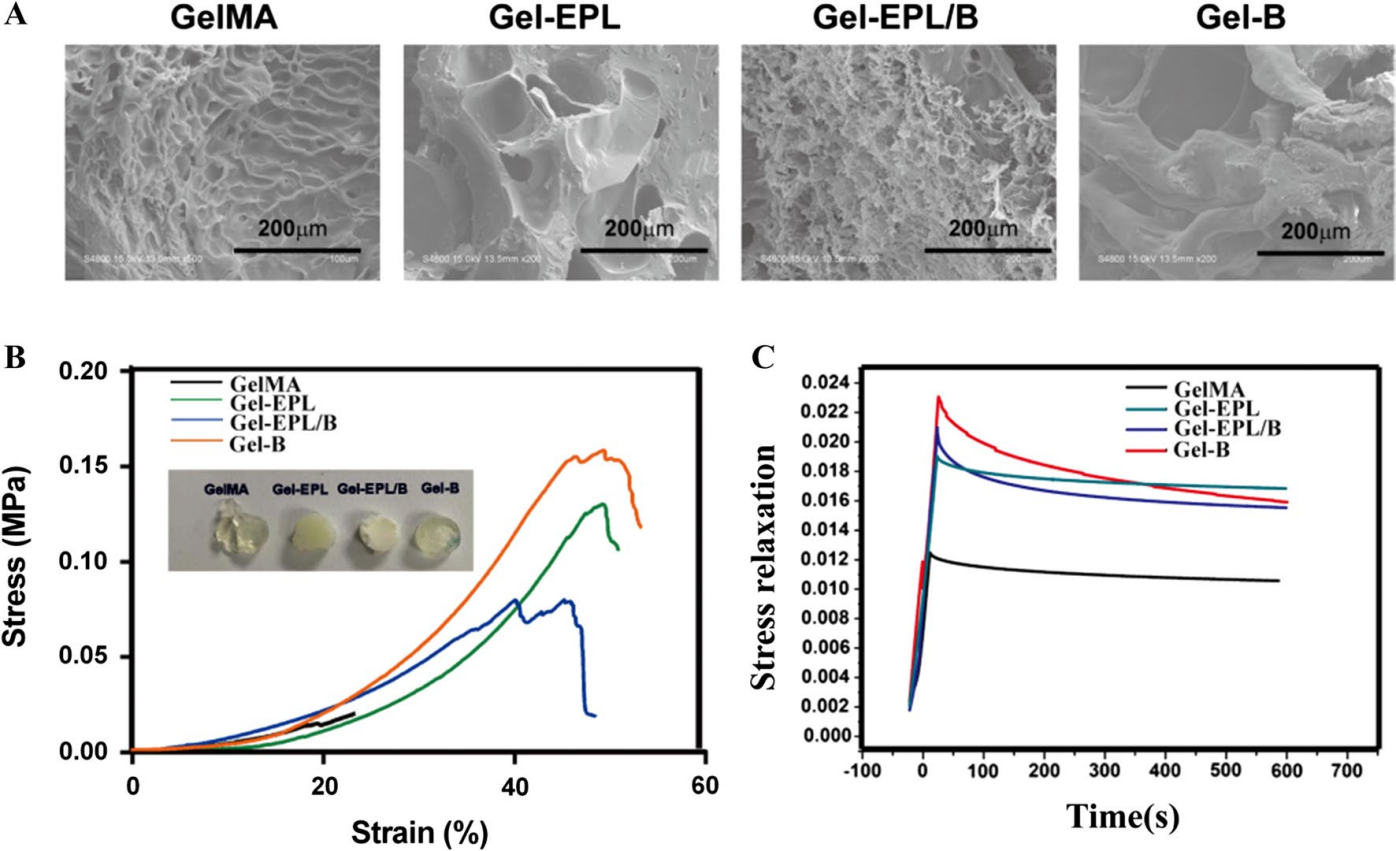
**A** SEM images of the four groups of GelMA hydrogels. **B** Representative stress–strain curves for different hydrogels. **C** Stress relaxation of different hydrogels

GelMA and Gel-B hydrogels were transparent, whereas EPL-modified hydrogels presented a white appearance (Fig. 2B). All hydrogels had a shorter gelation time, which was suitable for the preparation of injectable hydrogels (Fig. 2C). Considering the injectability and moldability of the hydrogel, GelMA-based hydrogel could be facilely injected, and rapidly modified to the desired shape (Additional file 1: Fig. S1F), and rendered to a rapid remolding. Equilibrium weight swelling ratios, gelation time, or mechanical behavior of these hydrogels were positively correlated with crosslinking density and type. EPL-modified hydrogels showed higher water absorption due to the reduction of the double bond. However, Gel-B hydrogel showed an insignificant decrease in swelling (Fig. 2D). Analysis of micromorphology of hydrogels by SEM showed that lyophilized hydrogels were highly porous, a property that is suitable for cell and tissue growth (Fig. 3A). Stress–strain testing showed that EPL-modified GelMA had greater toughness, fracture tensile ratio, and fracture strength compared with GelMA hydrogel (Fig. 3B). Gels from PBA-modified hydrogels showed faster stress relaxation compared with GelMA or Gel-EPL hydrogels. This finding implies that PBA-modified hydrogels had increased dynamic chemical bonds in the polymer network resulting in viscoelasticity of hydrogels thus increasing stress relaxation (Fig. 3C). Assessment of the degradation rates of the hydrogels allowed us to evaluate their biodegradation within 28 days in PBS containing Collagenase Type II (0.2 U/ml). Additional file 1: Fig. S1E shows that all hydrogels were degraded to more than 50% after 28 days. The Gel-EPL/B and Gel-B hydrogels exhibited a slower degradation rate than the GelMA hydrogel under the same conditions. The differences in degradation rates between the GelMA hydrogel and Gel-B hydrogel were attributed to the PBA chain, which protects cleavage sites on gelatin from collagenases.

### Biocompatibility of different hydrogels in vitro

**Fig. 4:**
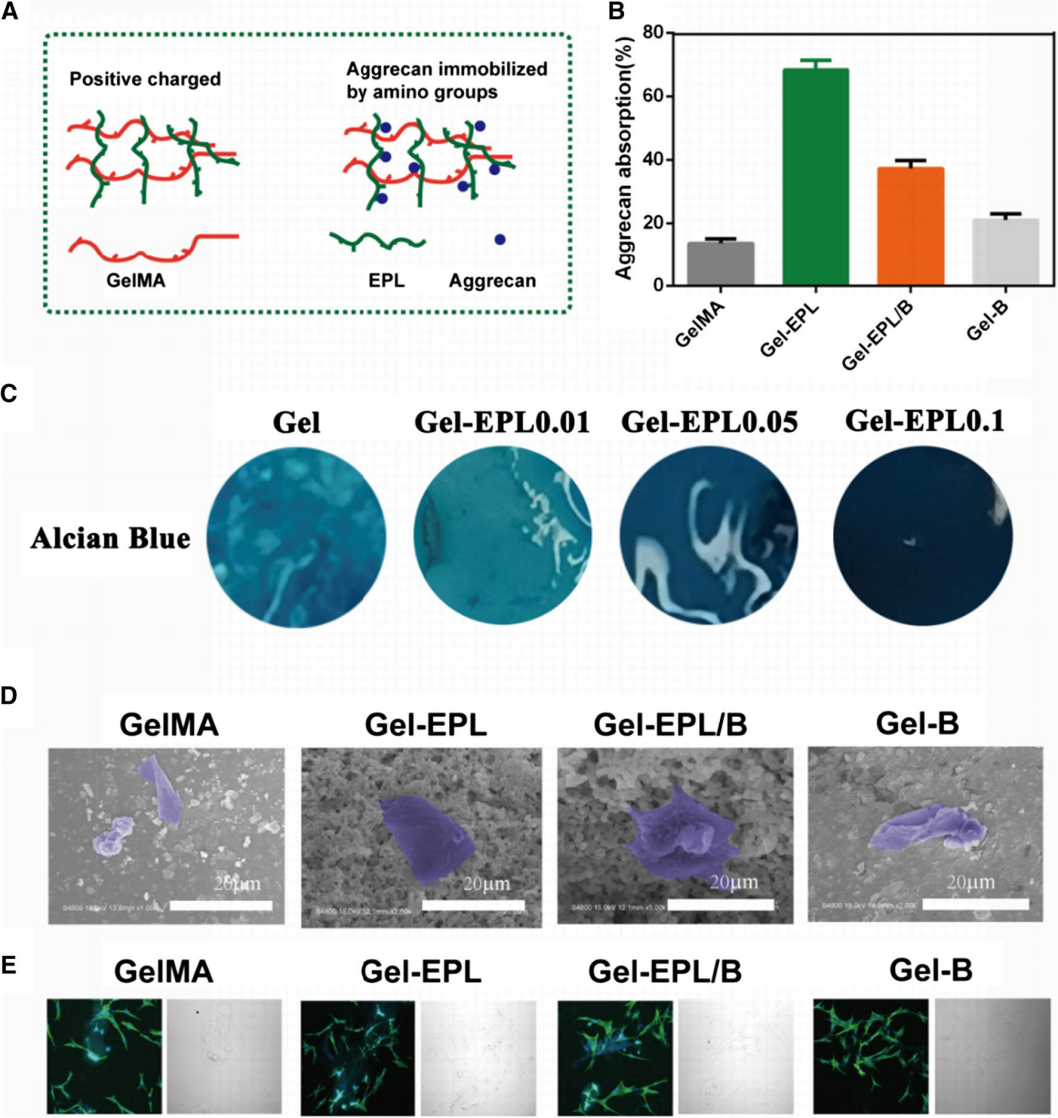
**A** Schematic diagram for immobilization of CS on the different surfaces of hydrogels. **B** Absorption of CS at a concentration of 0.5 mg/mL. **C** Alcian Blue stain represents acan absorption of different hydrogels from cell secretion by transwell. **D** SEM images of cells seeded for one day on different hydrogels. Cells on the hydrogels are marked in purple. **E** Cell adhesion on the surface of the hydrogel. F-actin (green) was visualized by FITC-phalloidin staining and nuclei (blue) were stained with DAPI. The figure on the right in **E** represents pictures in bright filed. Magnification: 400×

Cell adhesive property is important for regeneration materials [36]. SEM results showed excellent cell adhesion in all groups (Fig. 4D). Analysis of actin cytoskeleton organization, a key factor for understanding cell adhesion and mobility [37], showed well-oriented F-actin stress fibers in cells cultured on all hydrogels (Fig. 4E).

We also explored the cell growth on the four types of hydrogels using the MTT assay kit and viability of cells and the stability of the chondrocyte phenotype after different periods of blue light treatments. Col II were applied for demonstrating cell metabolic activity and stability of chondrocytes after the gel was irradiated with blue light (405 nm) for 1 min and then culture for 1 day and 3 days. As shown in Additional file 1: Fig. S2A, from 1 to 3 days, the number of chondrocytes increased, and chondrocytes in hydrogels still had strong extracellular matrix secretion ability. Additional file 1: Fig. S2B, C illustrates the proliferation characteristic of rBMSCs and chondrocytes. The cell viability absorbance values increased from day 1 to day 5 in the four groups, without any significant difference. These hydrogels could support chondrocytes and rBMSCs survival and proliferation.

### Surface immobilization capability of hydrogels

Surface chemistry affects protein absorption behavior. Therefore, we evaluated ECM immobilization properties of prepared hydrogels using Chondroitin sulfate (CS) models. CS, which is used for cartilage regeneration [38], contains sulfonic and carboxylic groups that exhibit negative charges. Immobilization on hydrogels is shown in Fig. 4A. Gel-EPL and Gel-EPL/B hydrogels exhibited positively charged surfaces, therefore, CS immobilization ability was significantly higher compared with GelMA and Gel-B hydrogels, which presented with negative charges (Fig. 4B). In addition, Gel-EPL could absorb more proteoglycans after 12 h of co-culture with chondrocytes, and staining became deeper with an increase in EPL (Fig. 4C).

### Biofunctionalization of hydrogels

**Fig. 5:**
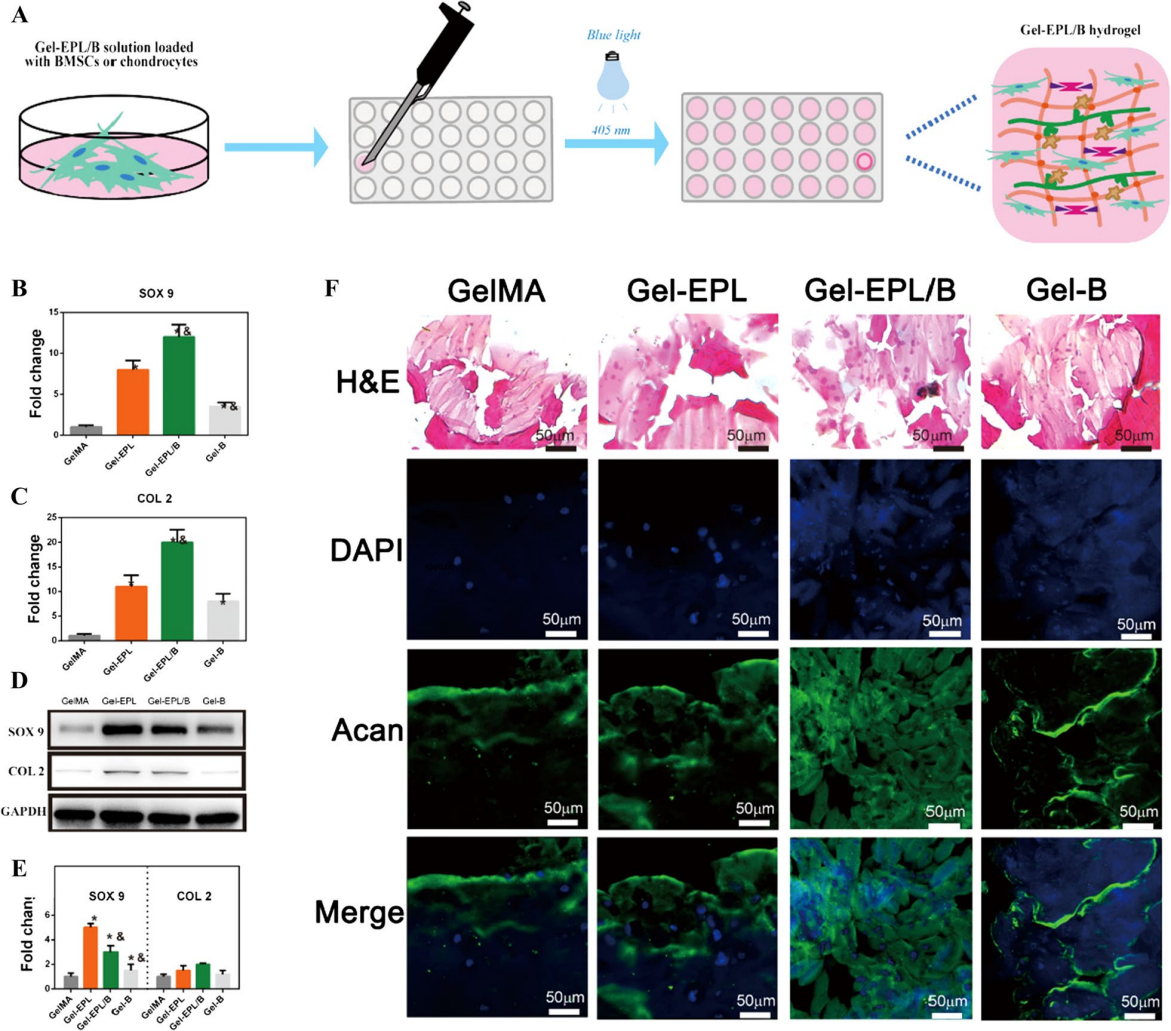
**A** The process of the three-dimensional culture of rBMSCs or chondrocytes in hydrogels. **B**, **C** Gene expression levels of SOX-9 (**B**) and COL II (**C**). **D**, **E** Western blot analysis of SOX 9 and Col II protein expression of rBMSCs seeded on different hydrogels. **F** Histology and immunofluorescence of GelMA and modified GelMA hydrogels after 2 weeks of culture

Expression levels of chondrogenesis genes including SOX-9, type II collagen (COL II) were evaluated to determine the chondrogenic differentiation potential of rBMSCs cultured in different hydrogels. The expression level of SOX-9 and collagen II from cells grown on Gel-EPL and Gel-EPL/B for 14 days was significantly higher compared with expression levels in cells grown on GelMA and Gel-B hydrogels (Fig. 5B, C). Western blot analysis showed high expression levels in cells cultured on Gel-EPL and Gel-EPL/B (Fig. 5D, E). ACAN is a key component for cartilage and markers, and chondrocytes were embedded in various hydrogels. During photo-crosslinking, the radicals generated are rapidly consumed in the crosslinking process, therefore cells are not adversely affected by blue light exposure during crosslinking. Production of extracellular matrix acan was observed after 2 weeks. Immunofluorescence staining showed a high expression level of acan in cells cultured on Gel-EPL and Gel-EPL/B (Fig. 5F).

### In vivo biocompatibility studies

**Fig. 6:**
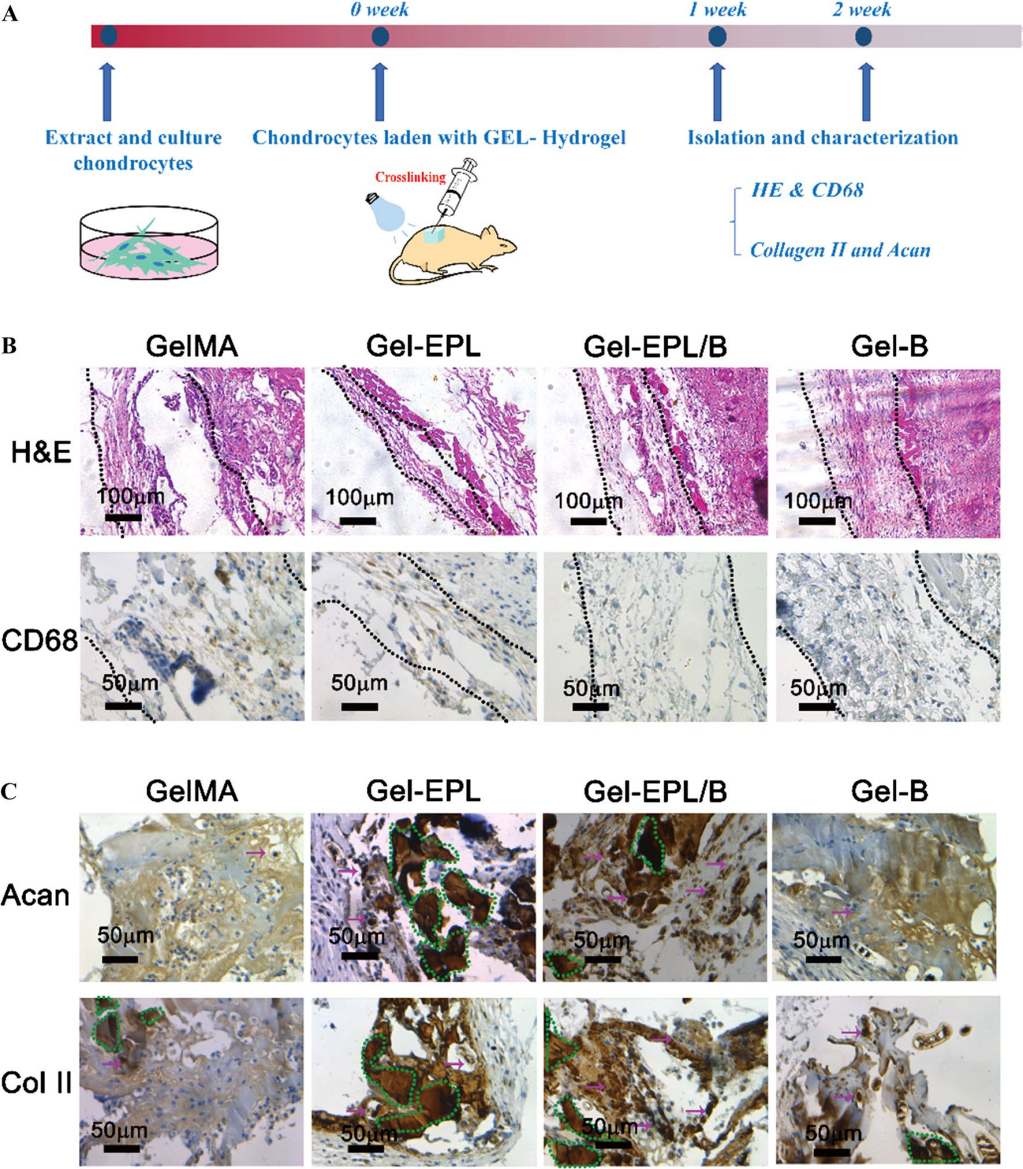
**A** The experimental procedure for injectable hydrogels for forming cartilage tissue in vivo. **B** In vivo biocompatibility and chondrocyte viability evaluation. Photomicrographs of H&E and CD68 showing GelMA and modified GelMA hydrogels subcutaneously implanted for 7 days. Dotted lines represent the extent of the fibrous capsules that enclose the materials. **C** In vivo evaluation of chondrocytes viability. The dotted circle in the figure represents the bulk materials and immunostainings (brown) showing cartilaginous matrix production in GelMA and modified GelMA hydrogels

High free amine concentrations can be cytotoxic [39–41]. Yuan *et. al* demonstrated modified porous PLGA microspheres with ε-poly-l-lysine (EPL) to promote cell growth on the microspheres. Cell experiments revealed that the cytotoxicity of microspheres was slightly increased post-EPL modification. However, their cell viability remained higher than 85% [40]. So, we firstly tested the biocompatibility of hydrogels in vivo (Fig. 6A). To assess in vivo biocompatibility of hydrogels, hydrogels were implanted subcutaneously into the back of mice. The tissue surrounding the implanted sites was isolated after 1 week and subjected to histology and immunohistochemistry (IHC) analyses. Samples were stained with H&E. Few signs of inflammation were observed in all groups. Inflammation and fibrous capsule thickness decreased significantly on Gel-EPL and Gel-EPL/B surfaces (Fig. 6B). Gel-EPL surface showed the least inflammation rate and fibrous capsule thickness (Fig. 6B).

### In vivo evaluation of chondrocyte viability

Cartilaginous tissue was formed after 2 weeks in both materials. However, more cartilage tissue was observed in the Gel-EPL/B group compared with the EPL-modified GelMA group as shown by the presence of high amounts of glycosaminoglycans using acan staining and by the presence of cartilage marker collagen type II (Fig. 6C). The formation of cartilaginous tissue implies that both tested materials are effective for cartilage tissue engineering purposes.

### Regeneration of cartilage defects in vivo

**Fig. 7:**
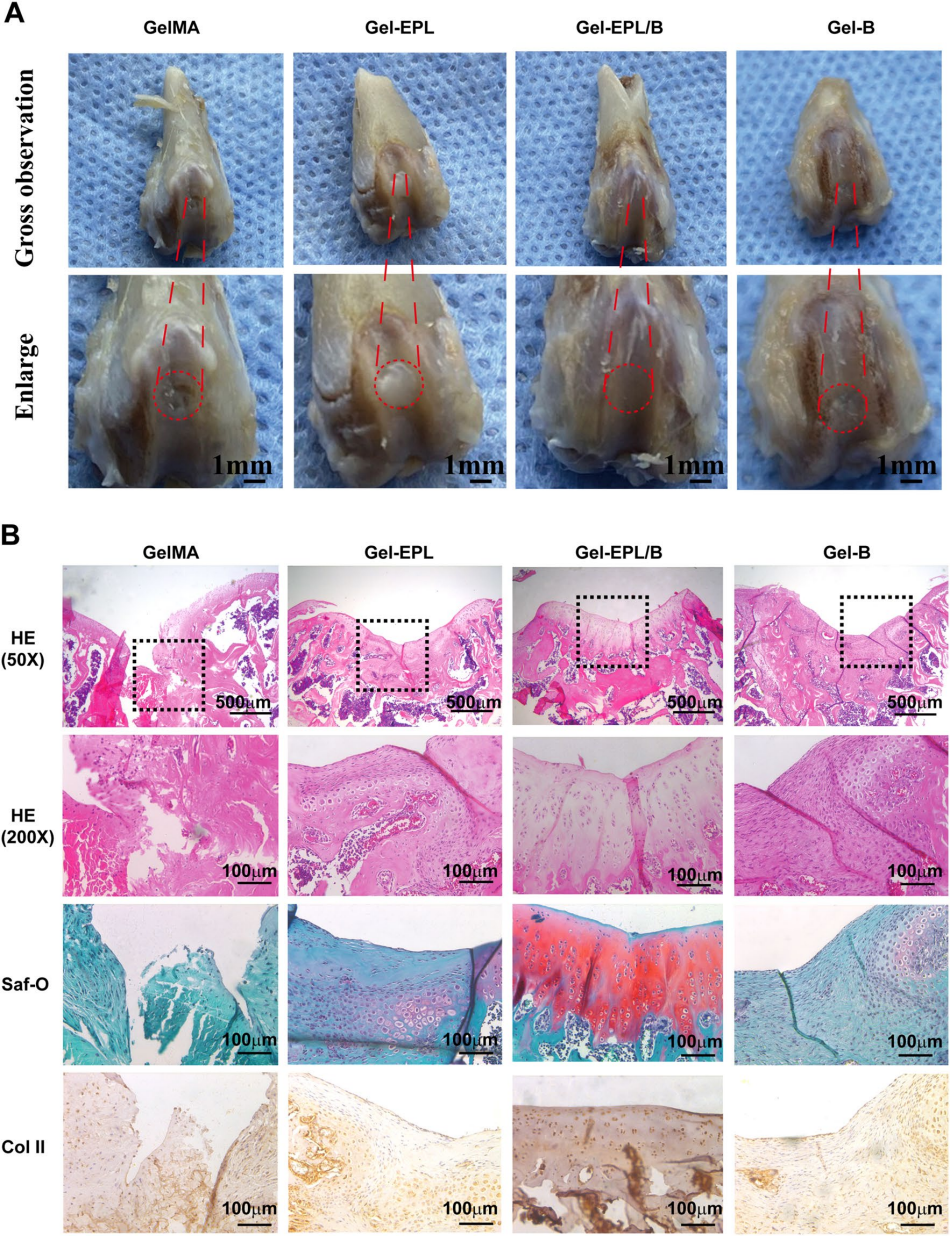
**A** Representative images of gross observation of cartilage defects treated with different hydrogels at 4 weeks post-operation in vivo in the rat. **B** Representative images of Hematoxylin and Eosin (HE) staining, Safranin-O (Saf-O) staining, and immunohistochemistry staining of type II collagen (Col-II) the cartilage defects and reparative tissues at 4 weeks post-implantation

In situ defect model was investigated to deeply elucidate the regenerative potential of the hydrogels in vivo. At 4 weeks post-operation, gross morphology examination demonstrated that the defect of the GelMA group was grossly distinguishable from adjacent cartilage tissue (Fig. 7A). However, the surface of the reparative tissue treated with modified GelMA hydrogels was at the same level as the adjacent cartilage. This denoted a higher degree of neo-tissue filling. Histologically, at 4 weeks, fibrous tissue was predominant in the GelMA group, with noticeable bone in-growth and absence of matrix staining for sGAG and Col-II (Fig. 7B). Comparatively, the defects in the Gel-EPL and Gel-B groups showed a significantly higher extent of neo-tissue formation with rounded chondrocytic cells and newly-synthesized matrix that stained strongly for sGAG and Col-II. Of note, Gel-EPL/B hydrogels were characterized by a smoother surface of repaired cartilage and a more comparable thickness to the native tissue. Moreover, the regenerated tissue displayed more uniform staining of GAGs and Col II compared to the GelMA hydrogels. This dually optimized scaffold may offer an ideal biomaterial for the regeneration of other joint cartilages.

## Discussion

Cell-based approaches in which chondrocytes are encapsulated in hydrogels are effective in cartilage regeneration [42–45]. For better regulation of cell behavior and tissue regeneration, natural and synthetic biomaterials have been designed [46]. Typically, biomaterials-engineering approaches focus on a few mechanisms (chemical or physical) by which ECM influences cells, and attempt to present these influences effectively for a given tissue. Here, we attempted to integrate the chemical cues and physical cues to explore the cartilage regeneration potential. Due to the strong interaction between EPL and negatively-charged molecules, EPL has been widely used in medical and tissue engineering fields [47, 48]. EPL is currently utilized as a food additive due to its biosafety. Previously, Hyon et al. reported a novel adhesive hydrogel that comprises dextran and epsilon-poly(l-lysine) (dextran-PL) and low cytotoxicity of the hydrogels [48]. The findings of our study showed that the number of amino groups in GelMA increases and the double bond of GelMA decreases with an increase in EPL. A higher EPL/GelMA ratio used in the pre-experiment showed that an increase in the ratio above 20% causes unable gelling (Data not shown) due to too few double bonds. Therefore, the maximum modification rate, the Gel/EPL 0.1 was chosen. Through the charge modification of the polymer chain, the Gel-EPL hydrogel prepared via our method can successfully adsorb negatively charged proteoglycan. Further cell experiments demonstrated that it potentially promoted the activity of chondrocytes and chondrogenic differentiation of stem cells. Current biomaterial strategies generally focus on the prevention of nonspecific protein adsorption and presentation of short biomimetic motifs, including RGD, to promote cell function [49, 50]. However, short biomimetic motifs always exhibit less biological activity compared to the native biomolecule. Therefore, developing synthetic surfaces to control the functional presentation of adsorbed bioactive moieties would offer an effective approach to precisely regulate cell-material biomolecular interactions. This will subsequently activate specific signaling pathways and differentiation programs for cartilage regeneration.

Beyond structural and biomechanical roles of hydrogels, physical properties also influence many aspects of cell behaviour. Several studies report that the mechanical properties of hydrogels affect cell behavior, adhesion, proliferation, deposition, and differentiation of extracellular matrix, ultimately affecting cell fate. A rigid hydrogel does not effectively promote communication between cells and the diffusion of new matrix [51]. Crosslinked gelatin hydrogel is stable and hard. Although mechanical properties are important for cell proliferation and differentiation support, they are not conducive for the generation of a new extracellular matrix and cell proliferation and communication in the later stage. This inhibits the formation of massive and complete cartilage implants. Although many studies report that the distribution and composition of the matrix of encapsulated chondrocytes are regulated by regulating the crosslinking density of the gel, detailed regulatory parameters have not been explored.

In this study, we introduced EPL into gelatin-based hydrogels and rapidly formed hydrogels under blue light. The hydrogels have high efficiency and innocuity, therefore, they can be used as injectables. In addition, prepared hydrogels showed good safety profiles. Positive charges were introduced to further improve biological activity, to optimize tissue regeneration. Moreover, the introduction of PBA ensured that the GelMA hydrogel was not hard and stubborn. Furthermore, PBA improved stress relaxation properties and promoted chondrocyte survival and differentiation of stem cells into chondrocytes. The results showed that the hydrogels were effective in promoting the differentiation of cartilage stem cells in vitro and in vivo.

In summary, this study provides information on the application of GelMA hydrogels in cartilage repair. Further, these findings provide a novel idea for exploring better cartilage repair scaffolds. For instance, we found that a combination of the chemical cues and physical cues specificity regulates the effects of surface chemistry on cartilage differentiation, and this establishes a mechanism for diverse cellular responses to biomaterial surface properties. Furthermore, this mechanism could be exploited to engineer materials that regulate the physical and chemical properties of materials to elicit desired cellular activities.

## Conclusion

In this study, modified gelatin (Gel-EPL, Gel-B, Gel-EPL/B) was synthesized from methacryloyl gelatin. The GelMA hydrogel, has injectable properties, good mechanical properties, and good biocompatibility in vivo and in vitro. Addition of several positive charges to EPL modified hydrogel promoted adsorption of negatively charged proteoglycans and secreted proteoglycans in the solution. Further, the hydrogel promotes deposition of the extracellular matrix of chondrocytes and chondrogenic differentiation of stem cells, thereby provides a good three-dimensional microenvironment for cartilage repair. Moreover, we introduced the dynamic covalent bond, which gave the hydrogel stress relaxation properties thus making the hydrogels more suitable for the mechanical properties of chondrocytes. The Gel-EPL/B hydrogel group promoted the formation of a large number of extracellular matrices. Moreover, chemical modification and mechanical modification demonstrated that hydrogel is effective in cartilage repair. This kind of artificial cartilage is analogous to natural cartilage in histology, molecular and mechanical properties. In summary, this modified hydrogel is a highly efficient material for chondrocytes which promotes cartilage repair.

## Supplementary Information

**Additional file 1:** **Fig. S1.** (A) Chemical structures of modified GelMA polymer,a-i represented H protons corresponding to ^1^HNMR spectrum. (B) ^1^H NMR spectra of Gel-EPL polymers with different EPL modification in D_2_O. (C) TG analysis indicating high stability of polymers above 300℃. (D) DSC curve of GelMA and modified GelMA polymers. (E) Degradation analysis of under enzymatic digestion with Collagenase Type II (0.2U/ml). (F) A demonstration of the injectability and moldability of GelMA -based hydrogels. **Fig. S2.** (A) Immunofluorescence staining Col II of chondrocytes with cultured within 3D Gel-EPL/B hydrogels over 1 and 3 days for demonstrating cell metabolic activity and stability of chondrocytes after the gel was irradiated with blue light (405nm) for 1min. (B-C) Proliferation of rBMSCs (C) and chondrocytes (D) cultured with hydrogels at the different time points was assessed by MTT assay.
