# Supplementary material for: Injectable stress relaxation gelatin-based hydrogels with positive surface charge for adsorption of aggrecan and facile cartilage tissue regeneration
Source: J Nanobiotechnology. 2021 Jul 18;19:214. doi: 10.1186/s12951-021-00950-0 (PMC8287687; doi:10.1186/s12951-021-00950-0)
Supplement: Supplementary file 1 — Additional file 1: Fig. S1. (A) Chemical structures of modified GelMA polymer,a-i represented H protons corresponding to 1HNMR spectrum. (B) 1H NMR spectra of Gel-EPL polymers with different EPL modification in D2O. (C) TG analysis indicating high stability of polymers above 300℃. (D) DSC curve of GelMA and modified GelMA polymers. (E) Degradation analysis of under enzymatic digestion with Collagenase Type II (0.2U/ml). (F) A demonstration of the injectability and moldability of GelMA -based hydrogels. Fig. S2. (A) Immunofluorescence staining Col II of chondrocytes with cultured within 3D Gel-EPL/B hydrogels over 1 and 3 days for demonstrating cell metabolic activity and stability of chondrocytes after the gel was irradiated with blue light (405nm) for 1min. (B-C) Proliferation of rBMSCs (C) and chondrocytes (D) cultured with hydrogels at the different time points was assessed by MTT assay. [file 12951_2021_950_MOESM1_ESM.doc]

Supporting Information

**Injectable Stress relaxation Gelatin-based Hydrogels with Positive Surface Charge for Adsorption of Aggrecan and Facile Cartilage Tissue Regeneration**

Kai-Yang Wang ^a #^, Xiang-Yun Jin ^b, #^, Yu-Hui Ma ^c, #^, Wei-Jie Cai^a^, Wei-Yuan Xiao^b^, Zhi-Wei Li^b^,*,Xin Qi^d^,*, Jian Ding^a^,*

Kai-Yang Wang, M.D., E-mail: [**ortho_wang@163.com**](mailto:ortho_wang@163.com)

Xiang-Yun Jin, M.D., E-mail: [**jinxiangyun920110@163.com**](mailto:jinxiangyun920110@163.com)

Yu-Hui Ma, M.D., E-mail: mayuhui_rehab@outlook.com

Wei-Jie Cai, Ph.D., E-mail: [**vegecai@alumni.sjtu.edu.cn**](mailto:vegecai@alumni.sjtu.edu.cn)

Wei-Yuan Xiao, M.D., E-mail: xwy2013_rjgk@hotmail.com

^a^ Department of Orthopedic Surgery, Shanghai Jiao Tong University Affiliated Sixth People’s Hospital, NO. 600, Yishan Rd., Shanghai, 200233, P.R.China.;

^b^ Department of Orthopedic Trauma, Department of Orthopedics, Renji Hospital, School of Medicine, Shanghai Jiao Tong University, Shanghai 200127, People's Republic of China

^c^ Department of Rehabilitation Medicine, Shanghai Jiao Tong University Affiliated Sixth People’s Hospital, NO. 600, Yishan Rd., Shanghai, 200233, P.R.China.;

^d^ Department of Orthopaedics, Shanghai Pudong Hospital, Fudan University Pudong Medical Center, No.2800 Gongwei Road, Huinan Town, Pudong, Shanghai, China

^#^ These authors contributed equally to this work.

***Corresponding Author:**

E-mail addresses: [leaf-in-wind271@hotmail.com (Zhi-Wei Li)](mailto:leaf-in-wind271@hotmail.com (Zhi-Wei Li))

[qixin19871012@163.com](mailto:qixin19871012@163.com) (Xin Qi)

[dingjian3246@163.com](mailto:dingjian3246@163.com) (Jian Ding)

**Supplemental Methods**

**Differential Scanning Calorimetry (DSC) Measurement.** The glasstransition temperature (Tg) of GelMA and modified GelMA polymers were determined by DSC (modulated DSC2910, 1090B, USA). The samples was carried out at a heating rate of 2℃/min from-50℃ to 60℃.

**Thermogravimetric Analysis (TGA).** Thermal degradation and stability measurements of polymers were performed using Thermogravimetric Analysis instruments (TGA, STA449F3) in a nitrogen atmosphere at a heating rate of 10 ℃ min^-1^ from 30℃ to 700℃.

**Degradation of hydrogels**

The *in vitro* degradation rate of the hydrogels was evaluated by soaking the samples in PBS containing Collagenase Type II (0.2U/ml) for 28 days at 37 ℃ after recording the dry weights of the initial (Wi) . At predetermined intervals, the samples were extracted, rinsed with deionized water, and lyophilized. The medium was refreshed each three days. We determined the degradation rate by recording the dry weights of the initial (Wi) and degraded (Wd) samples, as follows:

Degradation (%) =(Wi ―Wd)/Wi×100

**Immunofluorescence staining Col II of chondrocytes**

Three-dimensional culture of chondrocytes within Gel-EPL/B hydrogels and Col II were applied for demonstrating cell metabolic activity and stability of chondrocytes after the gel was irradiated with blue light (405nm) for 1min and then culture for 1 day and 3 days. Chondrocytes were mixed with hydrogel (100 μL, 20%, w/v) solution containing LAP (0.1%, w/v) at a concentration of 1×10^7^cells/mL.
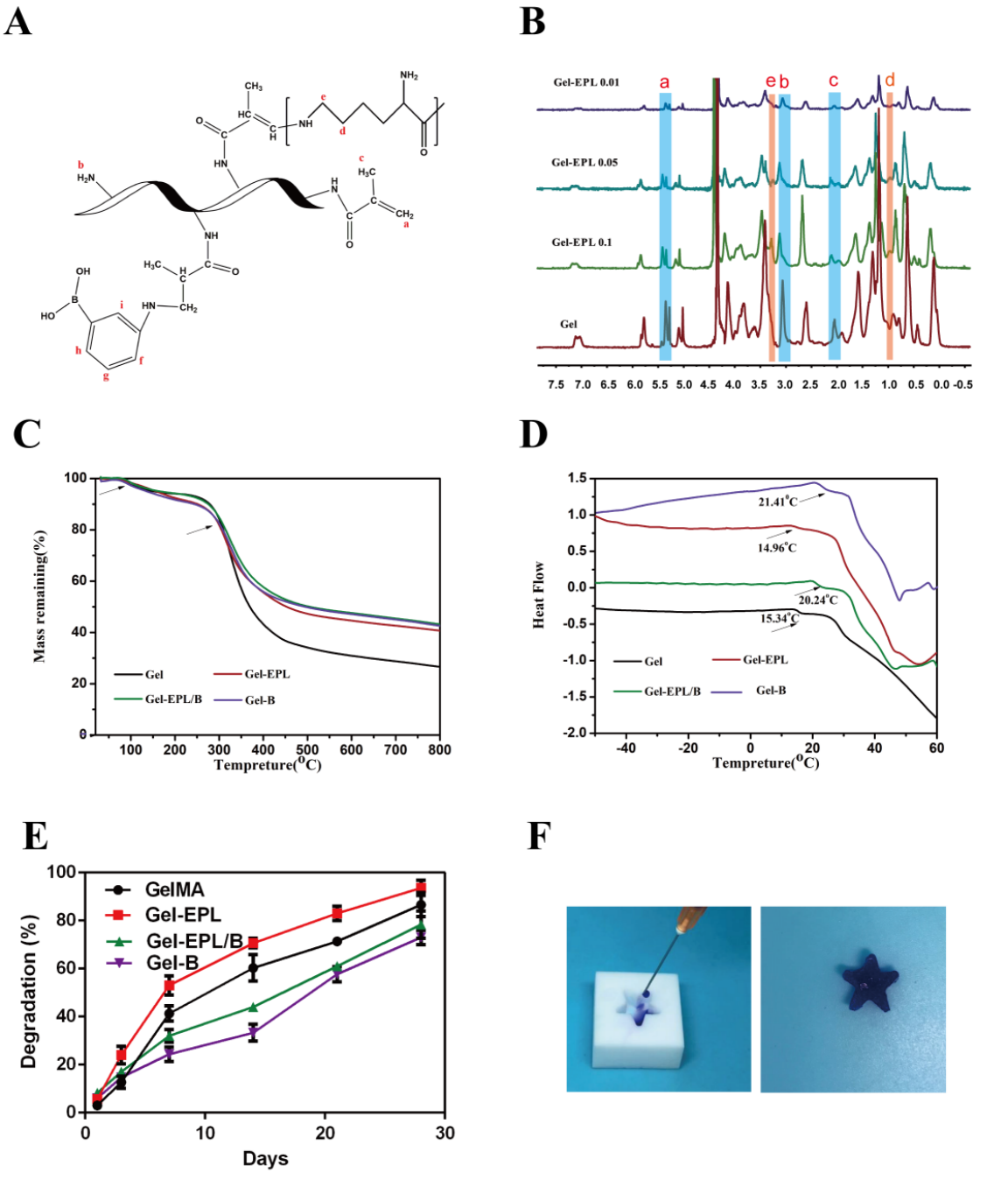


Fig.S1. (A) Chemical structures of modified GelMA polymer,a-i represented H protons corresponding to ^1^HNMR spectrum. (B) ^1^H NMR spectra of Gel-EPL polymers with different EPL modification in D_2_O. (C) TG analysis indicating high stability of polymers above 300℃. (D) DSC curve of GelMA and modified GelMA polymers. (E) Degradation analysis of under enzymatic digestion with Collagenase Type II (0.2U/ml). (F) A demonstration of the injectability and moldability of GelMA -based hydrogels.


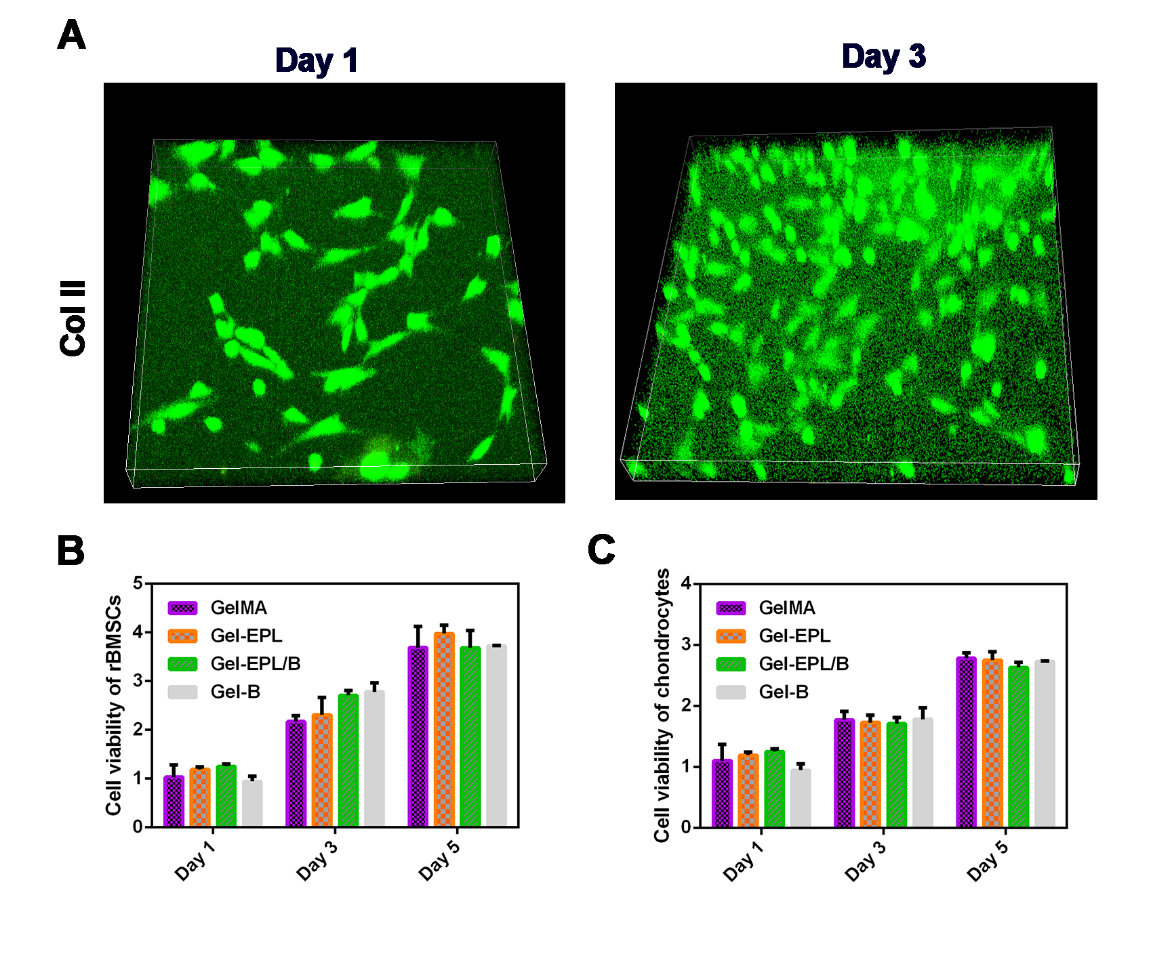


Fig.S2. (A) Immunofluorescence staining Col II of chondrocytes with cultured within 3D Gel-EPL/B hydrogels over 1 and 3 days for demonstrating cell metabolic activity and stability of chondrocytes after the gel was irradiated with blue light (405nm) for 1min. (B-C) Proliferation of rBMSCs(C) and chondrocytes(D) cultured with hydrogels at the different time points was assessed by MTT assay.
